# Elevated levels of circulating ITIH4 are associated with hepatocellular carcinoma with nonalcoholic fatty liver disease: from pig model to human study

**DOI:** 10.1186/s12885-019-5825-8

**Published:** 2019-06-25

**Authors:** Naohiko Nakamura, Etsuro Hatano, Kohta Iguchi, Motohiko Sato, Hiroaki Kawaguchi, Iwao Ohtsu, Takaki Sakurai, Nobuhiro Aizawa, Hiroko Iijima, Shuhei Nishiguchi, Takuya Tomono, Yukihiro Okuda, Seidai Wada, Satoru Seo, Kojiro Taura, Shinji Uemoto, Masaya Ikegawa

**Affiliations:** 1 Department of Surgery, Graduate School of Medicine, Kyoto University, Kyoto, Japan; 2 Department of Diagnostic Pathology, Graduate School of Medicine, Kyoto University, Kyoto, Japan; 3 Department of Surgery, Hyogo College of Medicine, Mukogowacho, Nishinomiya, Hyogo 663-8501 Japan; 4 Department of Hygiene and Health Promotion Medicine, Kagoshima University Graduate School of Medical and Dental Sciences, Kagoshima, Japan; 5 Department of Headquarters for International Industry-University Collaboration, Tsukuba University, Ibaragi, Japan; 6 Division of Hepatobiliary and Pancreatic Diseases, Department of Internal Medicine, Hyogo College of Medicine, Hyogo, Japan; 7 Department of Life and Medical Systems, Faculty of Life and Medical Sciences, Doshisha University, Kyoto, Japan

**Keywords:** Nonalcoholic fatty liver disease, Hepatocellular carcinoma, Pig model, Inter-alpha-trypsin inhibitor heavy chain 4, Blue native/SDS gel electrophoresis, MALDI-TOF MS/MS

## Abstract

**Background:**

Noninvasive biomarkers are urgently needed for optimal management of nonalcoholic fatty liver disease (NAFLD) for the prevention of disease progression into nonalcoholic steatohepatitis (NASH) and hepatocellular carcinoma (HCC). In order to identify the biomarkers, we generated the swine hepatocellular carcinoma (HCC) model associated with NAFLD and performed serum proteomics on the model.

**Methods:**

Microminipigs were fed a high-fat diet to induce NAFLD and a normal diet as the control. To induce HCC, diethylnitrosamine was intraperitoneally administered. Biopsied liver samples were histopathologically analyzed every 12 weeks. Serum proteins were separated by blue native two-dimensional gel electrophoresis and proteins of interest were subsequently identified by MALDI-TOF MS/MS. Human serum samples were analyzed to validate the candidate protein using antibody-mediated characterization.

**Results:**

In the NAFLD pigs, hepatic histology of nonalcoholic steatohepatitis (NASH) was observed at 36 weeks, and HCC developed at 60 weeks. Among serum proteins identified with MALDI-TOF MS/MS, serum inter-alpha-trypsin inhibitor heavy chain 4 (ITIH4), an acute response protein which is secreted primarily by liver, was identified as the most characteristic protein corresponding with NAFLD progression and HCC development in the NAFLD pigs. With immunoassay, serum ITIH4 levels in the NAFLD pigs were chronologically increased in comparison with those in control animal. Furthermore, immunohistochemistry showed ITIH4 expression in hepatocytes also increased in both the cancer lesions and parenchyma as NAFLD progressed. Human study is also consistent with this observation because serum ITIH4 levels were significantly higher in HCC-NAFLD patients than in the simple steatosis, NASH, and virus-related HCC patients. Of note, HCC-NAFLD patients who had higher serum ITIH4 levels exhibited poorer prognosis after hepatectomy.

**Conclusions:**

We established an HCC pig model associated with NAFLD. Serum proteomics on the swine HCC with NAFLD model implicated ITIH4 as a non-invasive biomarker reflecting NAFLD progression as well as subsequent HCC development. Most importantly, the results in the swine study have been validated in human cohort studies. Dissecting speciation of serum ITIH4 promises to have clinical utility in monitoring the disease.

**Electronic supplementary material:**

The online version of this article (10.1186/s12885-019-5825-8) contains supplementary material, which is available to authorized users.

## Background

Metabolic syndrome is now considered a major public health issue worldwide and the rising incidence of metabolic syndrome correlates with the high prevalence of nonalcoholic fatty liver disease (NAFLD) [1, 2]. NAFLD encompasses a clinicopathological spectrum of diseases ranging from simple to progressive steatosis, nonalcoholic steatohepatitis (NASH), and ultimately cirrhosis and hepatocellular carcinoma (HCC) [3]. The surge in the number of cases with NAFLD is projected to lead to an increase in the number of patients with NAFLD-related HCC [4]. The most worrisome issue is the onset of HCC in NAFLD patients who do not yet have cirrhosis [5]. At present, a liver biopsy is essential for the diagnosis of NASH and for prognostic risk stratification of NAFLD patients. Thus, noninvasive biomarkers indicating NAFLD activity over time that can be used as a prognostic factor for subsequent development of HCC are of great interest.

As is often the case with human biomarker studies, patients exhibit clinical heterogeneity based on habits, nutrition, comorbidities, and therapeutic interventions. In addition, the fact that HCC develops from NAFLD in humans over several decades presents difficulties in discovering serum biomarkers that reflect time-dependent processes of the disease. Many rodent models of NAFLD induced by genetic manipulations or diets have been developed [6]. However, these rodent models do not completely replicate the histological and clinical features of NAFLD in humans. By contrast, pigs are non-primate mammals that closely resemble humans in terms of anatomy, genetics, and physiology as well as lipid metabolism. As model organisms, they allow serial examinations to be conducted using the same experimental set [7, 8]. To generate a swine model of NAFLD associated HCC model, we adopted Microminipig (MMP) registered with the Japanese Ministry of Agriculture, Forestry and Fisheries as an experimental strain of swine. Recently, MMP was utilized as a model of atherosclerosis induced by feeding a high fat and high cholesterol diet, which means this strain must be as a useful animal model for understanding the pathophysiology of a variety of metabolic syndrome [9].

In recent years, mass spectrometry-based proteomics has been utilized for biomarker research in various diseases. Many proteins in the blood are synthesized in the liver, and the abundance and structure of many of these proteins change in response to the longitudinal course of liver diseases, including NAFLD [10, 11]. Thus, understanding the blood proteome corresponding to the liver pathology of different stages of the disease has the potential to provide unique information about disease-associated biomarker proteins. Although numerous biomarkers for NAFLD have been identified by proteomic techniques using animal models, there are few biomarkers that have validated the clinical utility in human cohorts [12]. On the other hand, established clinical scoring system for NAFLD progression could predict advanced fibrosis but not development of HCC [13].

Using a successfully established swine model of NAFLD associated HCC on MMPs, we have found a significant alteration of serum proteome in terms of multiple protein complex (MPC) formation as disease progress. Among those proteins, inter-alpha-trypsin inhibitor heavy chain 4 (ITIH4) plays an important role in predicting the course of NAFLD over time, from simple steatosis to NASH and HCC. Most importantly, the current swine study was validated with human studies to show that serum ITIH4 can be a robust and an expecting biomarker of the disease.

## Methods

### Animals and diets

Three male MMPs aged 3–4 months were purchased from Fuji Micra, Inc. (Shizuoka, Japan). In NAFLD group, two pigs were fed a high-fat diet (HFD) in which 47% of the total calories were from fat, 21.5% calories from fructose, and 16% calories from protein (Research Diets, New Brunswick, NJ, USA). In the HFD, methionine and choline were provided at 3500 and 822 ppm concentrations, respectively. The diet also consisted of 2% cholesterol and 0.7% sodium cholate by weight The third pig was fed a normal diet at 3% of body weight per day as a control animal. The normal diet was composed of less than 10.0% carbohydrate, greater than 13.0% protein, and 2.0% fat by weight (Marubeni Nisshin Feed Inc., Tokyo, Japan). The three pigs were maintained at 24 ± 3 °C under a 12-h light/dark cycle with free access to water.

### Experimental protocol

Twelve weeks after starting dietary intervention, two pigs that were fed the HFD were intraperitoneally administered diethylnitrosamine (DEN; 60 mg/kg body weight) every 2 weeks until 36 weeks. On the contrary, a pig that was fed a normal diet and was not administered DEN was used as the control. Body weight, abdominal girth, and blood pressure were measured every 4 weeks. Blood pressures were measured at the foreleg using the Manschette method. Blood analysis and liver biopsies were performed every 12 weeks after 12 h of fasting from the last feeding. Blood samples were collected from the sinus venarum cavarum. At liver biopsies, pigs underwent laparotomy with mid-line incision under general anesthesia. Gross observation of the whole liver was performed in order to check for the presence or absence of tumor development. Liver tissues were incised after the addition of transfixation ligatures with a 3–0 braid absorbable suture on the line just proximal to a scheduled cutting line. A portion of each sample was fixed with 10% formaldehyde and embedded in paraffin wax. Another portion was snap-frozen in liquid nitrogen immediately after sampling. All experiments and measurements were performed under general anesthesia. As premedication for general anesthesia, animals were sedated with intramuscular medetomidine hydrochloride (0.08 mg/kg body weight), midazolam (0.08 mg/kg body weight), and atropine sulfate (0.03 mg/kg body weight). General anesthesia was induced and maintained by inhalation of 1–2% isoflurane. Pulse oximetry and noninvasive blood pressure were monitored during experiments. The physical conditions of the animals were checked every day. If abnormal symptoms such as general fatigue, decreased activity, frequent vomiting, or respiratory distress were observed, pigs were killed before the predetermined day. After 60 weeks, the pigs were killed by cutting the inferior vena cava under deep anesthesia using isoflurane. All protocols were approved by the Ethics Committee of Animal Care and Experimentation at Kyoto University (MedKyo16619).

### Serum biochemistry

10 ml of serum was separated by centrifugation at 3500 rpm for 10 min and stored at − 80 °C immediately. Serum triglyceride (TG), total cholesterol (TC), low-density lipoprotein cholesterol (LDL-C), high-density lipoprotein cholesterol (HDL-C), and blood sugar levels were measured using standard methods. Serum liver biochemical parameters were also measured by a local clinical laboratory.

### Histological assessment of NAFLD activity and tumor characteristics

Paraffin-embedded tissues were sectioned at a thickness of 4 μm and stained with hematoxylin and eosin or Masson trichrome stain. Pathologists reviewed stained glass slides to provide NAFLD activity scores (NAS). The NAS was created as an unweighted score for steatosis (0–3), lobular inflammation (0–3), and ballooning (0–2). In addition, fibrosis was described as stage 1 to 4 [14]. Based on these results, the samples at each time point from the three pigs were divided into four groups according to total NAS and presence or absence of HCC; NAS = 0, NAS = 1–3, NAS ≥ 4 without HCC, and NAS ≥ 4 with HCC group.

### Immunohistochemistry

After deparaffinization and rehydration, antigen retrieval was applied by microwaving for 10 min with 0.01 M citric acid buffer. The endogenous peroxidase was destroyed by hydrogen peroxide. Primary antibodies were reacted at 4 °C overnight. The secondary antibody (EnVision System-HRP; Dako, Glostrup, Denmark) was incubated for 1 h at room temperature. Subsequently, staining with 3,3′-diaminobenzidine (Dako) for 3 min was performed. For immunohistochemistry of tumor lesions, rabbit polyclonal antibodies for GS (11037–2-AP; Proteintech, Chicago, IL, USA; 1:200 dilution), GPC3 (ab66596; Abcam, Cambridge, UK; 1:200 dilution), HSP70 (10995–1-AP; Proteintech; 1:200 dilution), and ARG1 (AV45673; Sigma-Aldrich, Steinheim, Germany; 1:200 dilution) were used as primary antibodies.

### Two-dimensional blue native/SDS gel electrophoresis (2D BN/SDS-PAGE)

The 2D BN/SDS-PAGE permits high-resolution separation of the serum MPC. First, 2 μl of serum was placed on a centrifugal filter column (Amicon Ultracel 3 K; Millipore, Billerica, MA, USA) with 500 μl of blue native buffer. The buffer consisted of 6-aminohexanoic acid, 200 mM Bistris, 500 mM EDTA, 5 M NaCl, and 20% glycerol. After centrifugation for 90 min at 15,000 rpm at 4 °C, the column was inverted in a microcentrifuge tube and centrifuged to collect the sample. After the preparation of serum, samples were mixed with BN loading buffer (2% (w/v) Coomassie 250 G and 750 mM aminocaproic acid: 10% of the sample volume). In the first-dimensional BN-PAGE, 12 μl of sample was applied per lane to a 4 to 15% gradient gel (Bio-Rad, Hercules, CA, USA), and electrophoresis was performed at 50 to 80 V at 4 °C with BN running buffer (25 mM Tris and 192 mM glycine). Next, the first-dimension gel was incubated for 30 min in reducing solution (20% (v/v) glycerol, 25% (v/v) 1 M Tris, and 1% (w/v) dithiothreitol). For further separation in the second dimension with sodium dodecyl sulfate (SDS)-PAGE, the lanes from the first-dimension gel were cut out and placed onto 12% gels (Bio-Rad) at the position of the teeth of a normal gel comb. The SDS-PAGE was performed at 100 V for 100 min at room temperature with SDS running buffer (25 mM Tris, 192 mM glycine, and 0.1% (w/v) SDS). After completion of gel electrophoresis, analytical gels were stained with colloidal Coomassie blue to visualize protein spots.

### Matrix-assisted laser desorption ionization-time of flight tandem mass spectrometry (MALDI-TOF MS/MS)

Protein spots excised from 2D gels were dehydrated with acetonitrile and dried by centrifuge for 20 min. The gel pieces were rehydrated in 10 μl trypsin (Roche, Mannheim, Germany) solution (5 μg/ml in 50 mM NH4HCO3 and 5 mM CaCl2) on ice for 45 min and then incubated at 37 °C overnight. Peptides were extracted twice using 5% HCOOH in 50% acetonitrile and dried. Digested peptides were dissolved in 0.1% tri-fluoroacetic acid and desalted using a C18 ZipTip (Millipore). The eluted peptide was applied to a MALDI target plate (MTP AnchorChip; Bruker Daltonik, Bremen, Germany) with 5 mg/ml α-cyano-4-hydroxycinammic acid (Bruker Daltonik) dissolved in 0.1% tri-fluoroacetic acid and 50% acetonitrile and was dried at room temperature. The target plate was mounted onto an AutofleX II device (Bruker Daltonik), and MALDI-TOF MS was performed in the positive ion reflector 1–3 kDa mode. Several precursor peaks with signal-to-noise ratios > 5 from each MS spectrum were selected manually and subjected to tandem mass spectrometry (LIFT-TOF/TOF) analysis. The generated mass spectra were identified by searching the database with the MASCOT server 2.3 (Matrix-Science, Boston, MA, USA). Mascot search conditions were as follows; Database: SwissProt, Taxonomy: Mammalia, Enzyme: trypsin, Global Modification: Carbamidomethyl (C), Variable Modification: Oxidation (M), tolerance: 300 ppm in MS, 0.8 Da in MS/MS.

### Western blotting

To validate the MS/MS results, we performed western blotting analyses using sera from both groups from 0, 12, 24, 36, 48, and 60 weeks. 2 μl of each sample applied per lane of a 4 to 15% gradient gel (Bio-Rad), and electrophoresis was performed at 50 to 80 V at room temperature with SDS loading buffer. The gels were transferred onto polyvinylidenedifluoride membranes in transfer buffer (25 mM Tris, 192 mM glycine, 0.01% (w/v) SDS, and 20% (v/v) methanol) at 12 V for 1 h. After blocking with 5% skim milk in phosphate-buffered saline for 1 h, the membranes were probed with a primary antibody overnight at 4 °C followed by horseradish peroxidase-conjugated secondary antibody (Cell Signaling Technology, Danvers, MA, USA) for 1 h at room temperature. Signals were detected using a Chemiluminescence Kit (Chemi-Lumi One; Nacalai Tesque, Kyoto, Japan) and visualized by the digital imager. Rabbit anti-ITIH4 antibody (24069–1-AP; Proteintech, Chicago, IL, USA; 1:1000 dilution), anti-ceruloplasmin (Cp) antibody (ab110449; Abcam, Cambridge, UK; 1:1000 dilution), and anti-haptoglobin (Hpt) antibody (NBT-MFG-102; Cosmo Bio Co., Ltd., Tokyo, Japan; 1:1000 dilution) were used as primary antibodies.

### Enzyme-linked immunosorbent assay (ELISA)

ELISA tests were used to quantify the protein concentration of ITIH4 in serum samples at all time points in both groups. The assay was carried out using an ITIH4 ELISA kit based on the sandwich ELISA principle (LifeSpan BioSciences, Seattle, WA, USA). 100 μl of serum sample was applied per well and incubated at 37 °C for 2 h. Biotinylated detection antibody was reacted at 37 °C for 1 h, and avidin-horseradish peroxidase conjugate was then added. Color development was induced by the addition of 3,3′,5,5′-tetramethyl benzidine substrate. The optical density of the well was measured at a wavelength of 450 nm.

### Immunohistochemistry of the liver

Expression levels of ITIH4 protein in the liver were analyzed by immunohistochemistry. Immunohistochemistry was performed using a rabbit anti-ITIH4 antibody (Proteintech; 1:200 dilution) in the liver tissues of NAFLD and control pigs at 0, 12, 36, and 60 weeks.

### Human cohort study

We obtained serum samples from four clinically classified groups. The HCC with NAFLD group included 55 HCC patients associated with NAFLD who underwent hepatectomy for HCC at Kyoto University Hospital between January 2007 and December 2016. These patients were characterized as negative for hepatitis C virus (HCV) antibody, hepatitis B surface antigen, and chronic alcohol consumption. For the simple steatosis (SS) group, we used serum samples from 40 liver transplantation donors who were diagnosed as SS by histological examination. They did not have a history of chronic alcohol consumption. For the NASH group, serum samples were obtained from 40 NASH patients without HCC who underwent liver biopsy and were diagnosed as NASH by pathologists at Hyogo College of Medicine Hospital. In addition, 21 HCV and 14 hepatitis B virus (HBV)-related HCC patients who underwent hepatectomy between January 2015 and December 2016 were included in the virus-related HCC group. All samples were collected before surgery and stored at − 80 °C until use. Using 2 μl of each serum sample, western blotting was performed with anti-ITIH4 antibodies (sc-515,353; Santa Cruz Biotechnology, Santa Cruz, CA, USA; 1:1000 dilution). The expression level of ITIH4 was quantified using average pixel intensity by densitometry. The diagnostic performance of ITIH4 intensity regarding NAFLD progression and HCC development was evaluated by multivariate receiver operating characteristic (ROC). In order to assess the relationship between serum ITIH4 levels and patients’ prognosis, 55 patients in the HCC with NAFLD group were divided into the low ITIH4 and high ITIH4 groups and their overall survival (OS) was compared. This study was approved by the Ethics Committee of Kyoto University Graduate School and Faculty of Medicine (approval code: R0261–1).

### Statistical analyses

Categorical variables were compared by Pearson’s chi-squared test. Continuous variables were compared using Student’s *t*-tests. OS curves for the low ITIH4 and high ITIH4 groups were estimated using the Kaplan-Meier method, and the results were examined using the log-rank and Wilcoxon tests. With regard to OS, univariate and multivariate hazard ratios (HR) were estimated using the Cox’s hazards regression model. All *P*-values were two-sided, and differences with a *P* <  0.05 were considered statistically significant. All statistical analyses were performed using JMP 8.0 software (SAS Institute, Cary, NC, USA).

## Results

### Physical characteristics and serum biochemical parameters of the animal model

Pigs in the NAFLD group exhibited greater weight gain than the control pig. Body weight in the NAFLD group increased to nearly 35 kg, but that in the control pig was under 23 kg at 48 weeks. Systolic blood pressure in the NAFLD group also elevated at the 48 weeks (Table 1). The NAFLD group showed aortic atherosclerotic lesions at the end of experimental period (Additional file 1: Figure S1). In terms of serum biochemistry, the NAFLD group exhibited severe hypercholesterinemia with high LDL-C levels around 24 weeks. TC and LDL-C levels in the control animal remained low during the experimental period. Blood sugar levels in the NAFLD group were elevated at 60 weeks (Table 1).

**Table 1:** Changes in body characteristics and serum biochemical parameters over time

weeks		0	12	24	36	48	60
Body weight (kg)	NAFLD No. 1	4.0	7.1	15.1	25.6	34.9	41.1
	NAFLD No. 2	5.2	12.2	21.0	33.5	35.5	33.7
	Control	6.2	9.6	13.9	18.3	22.7	25.4
Abdominal circumference (cm)	NAFLD No. 1	39	46	63	76	81	90
	NAFLD No. 2	40	55	71	75	83	81
	Control	45	51	62	66	70	74
Systolic blood pressure (mmHg)	NAFLD No. 1	105	124	122	120	166	158>
	NAFLD No. 2	91	133	116	130	134	134
	Control	89	105	87	100	114	114
TC (mg/dl)	NAFLD No. 1	84	366	449	201	181	214
	NAFLD No. 2	75	485	600	704	372	249
	Control	66	76	107	76	52	63
LDL-C (mg/dl)	NAFLD No. 1	33	81	122	60	45	96
	NAFLD No. 2	40	225	112	291	143	118
	Control	31	23	21	24	17	19
HDL-C (mg/dl)	NAFLD No. 1	47	120	108	82	90	72
	NAFLD No. 2	30	75	144	105	95	46
	Control	34	52	80	51	34	42
TG (mg/dl)	NAFLD No. 1	5	7	21	6	6	12
	NAFLD No. 2	5	10	13	15	10	6
	Control	8	16	13	27	11	12
BS (mg/dl)	NAFLD No. 1	72	83	89	100	70	124
	NAFLD No. 2	85	115	118	122	113	136
	Control	73	84	44	86	91	65

*TC* Total cholesterol, *LDL-C* Low density lipoprotein cholesterol, *HDL-C* High density lipoprotein cholesterol, *TG* Triglycerides, *BS* Blood sugar

### Serial liver biopsies demonstrate progressive NASH histological changes in NAFLD

**Fig. 1:**
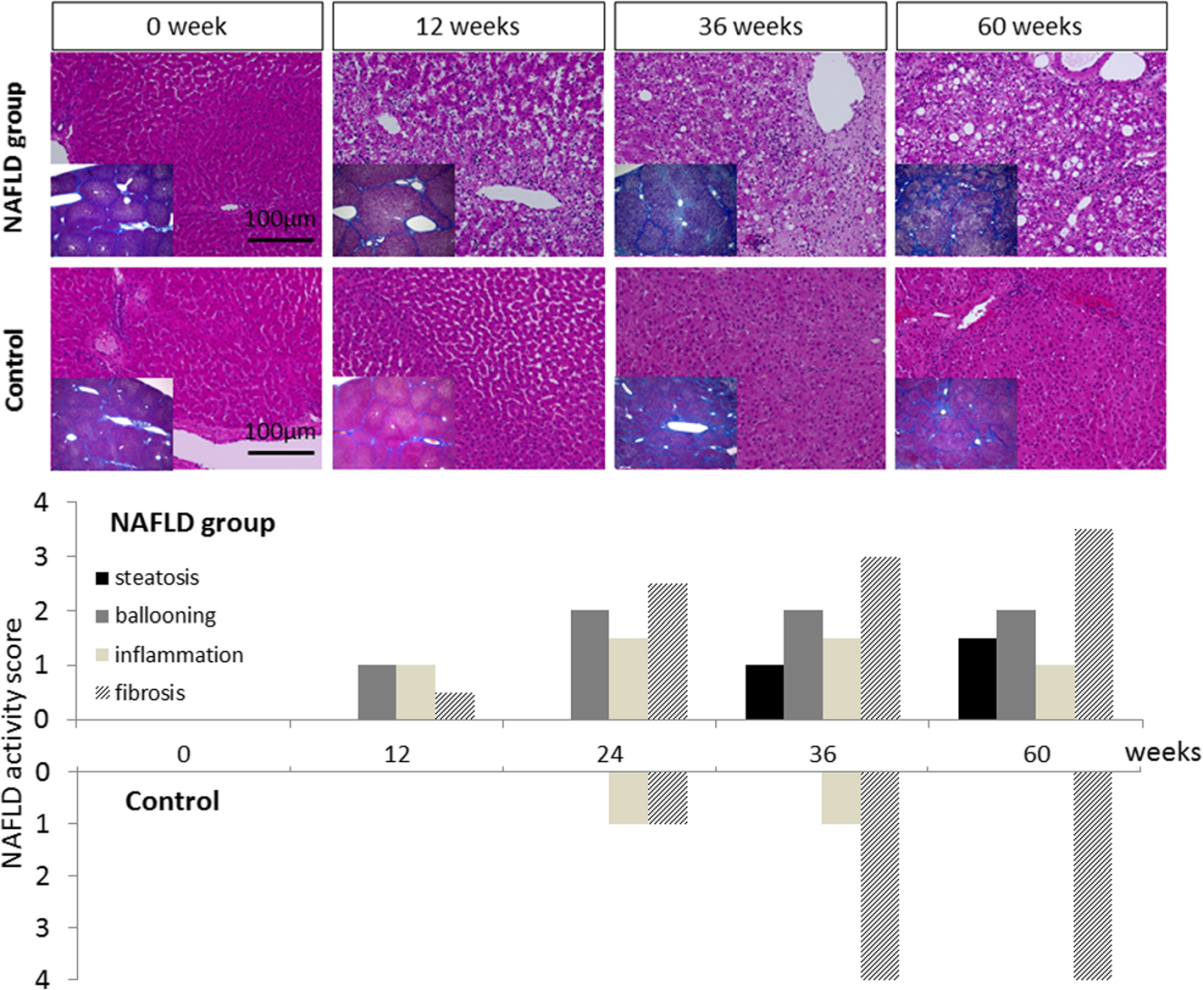
Changes in histological findings and NAFLD activity scores over time in the NAFLD group and control. Hematoxylin and eosin stained sections of samples from the NAFLD group (× 200) revealed that hepatocyte ballooning and lobular inflammation with lymphocyte infiltration appeared in the perivenular region (zone 3) at 12 weeks and became diffuse at 36 weeks. Microvesicular steatosis also appeared at 36 weeks. Masson trichrome staining (× 20) revealed slight fibrosis in zone 3 at 12 weeks; pericellular fibrosis, at 36 weeks; and complete bridging fibrosis, at 60 weeks. In the control animal, lobular inflammation was observed at 24 and 36 weeks. Fibrosis appeared in zone 3 at 24 weeks, and cirrhosis developed at 36 weeks. NAFLD activity scores in the NAFLD group are expressed as mean values. The NAFLD group; HFD feeding with DEN injection. The control; normal diet feeding without DEN injection

In the NAFLD group, hepatocyte ballooning and lobular inflammation started to appear at 12 weeks and became diffuse at 36 weeks (Fig. 1). Microvesicular steatosis was also observed at 36 weeks. Fibrosis started to appear at 12 weeks and progressed until 60 weeks. The average of total NAS gradually increased and reached 4.5 points at 36 weeks. In the control animal, steatosis was not observed during the experiment. However, fibrosis appeared at 24 weeks, and cirrhosis developed as early as 36 weeks.

### Multiple HCC is reproducibly observed at 60 weeks in NAFLD group

**Fig. 2:**
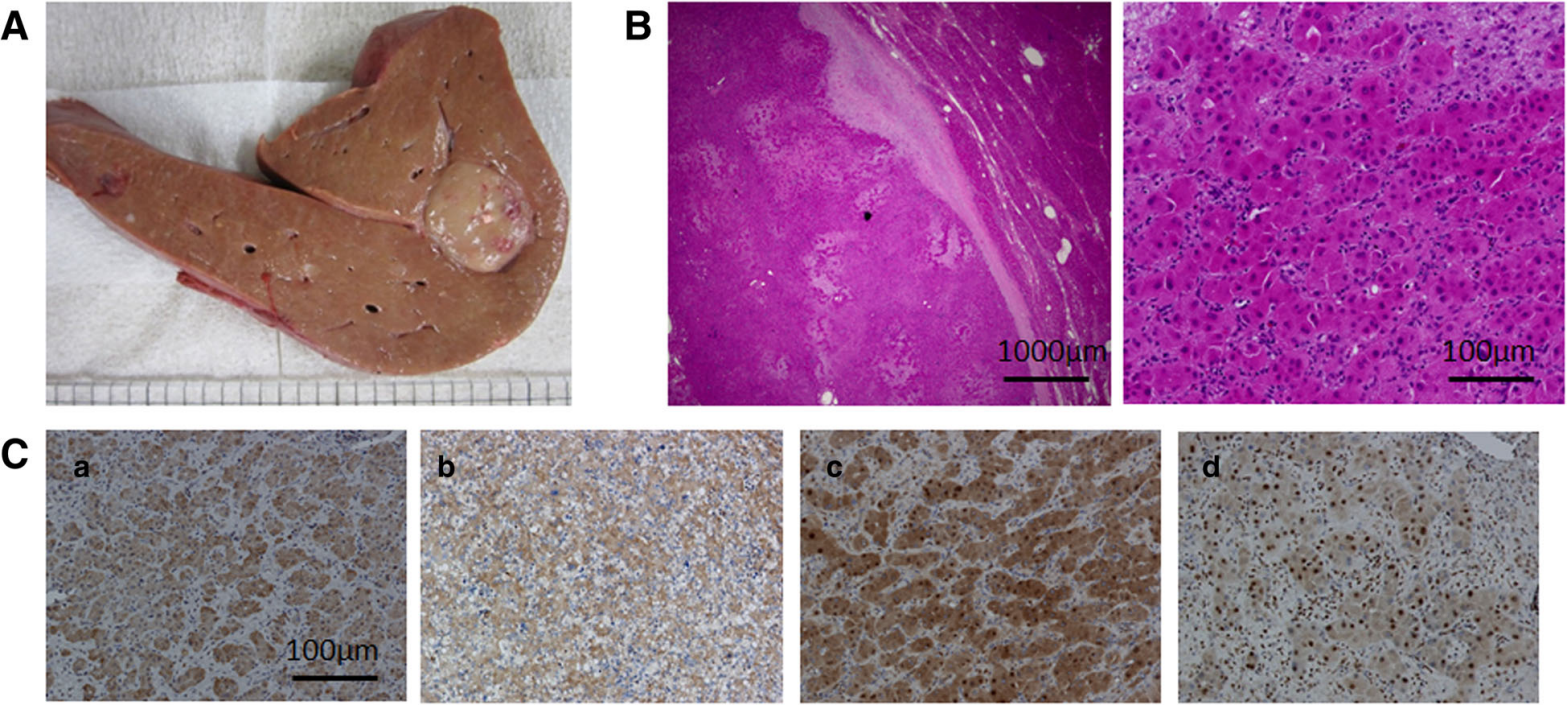
Histological features of liver tumors in the NAFLD group. (**a**) Macroscopic view of the liver in the NAFLD group. All animals in the NAFLD group developed multiple liver tumors at 60 weeks. Encapsulated tumor nodules with various sizes were observed in the liver. (**b**) Histological features of tumor lesions with hematoxylin and eosin staining. Tumor lesions compressed the surrounding liver parenchyma and exhibited fatty changes (a: × 20). Cellular atypia with large nuclei, small cells, and trabecular structures were observed in the tumor lesions (b: × 200). (**c**) Immunohistochemistry for well-differentiated HCC markers in tumor lesions. Glutamine synthetase showed diffuse cytoplasmic staining (a: × 200). Glypican-3 showed faint staining (b: × 200). Heat-shock-protein 70 showed strongly diffuse nucleo-cytoplasmic staining, not only in the tumor lesions but also in the non-tumor lesions (c: × 200). Arginase-1 showed focal nucleo-cytoplasmic staining (d: × 200)

Multiple encapsulated liver tumors developed at 60 weeks in both pigs in the NAFLD group (Fig. 2A). However, the control pig did not exhibit any liver tumors at 60 weeks. The tumor lesions exhibited cellular atypia with large nuclear, trabecular structures, and fatty changes (Fig. 2B) and showed immunoreactivity for GS, GPC3, HSP70, and ARG1 (Fig. 2C).

### Characteristic serum MPC profile of NAFLD with HCC identified by 2D BN/SDS-PAGE

**Fig. 3:**
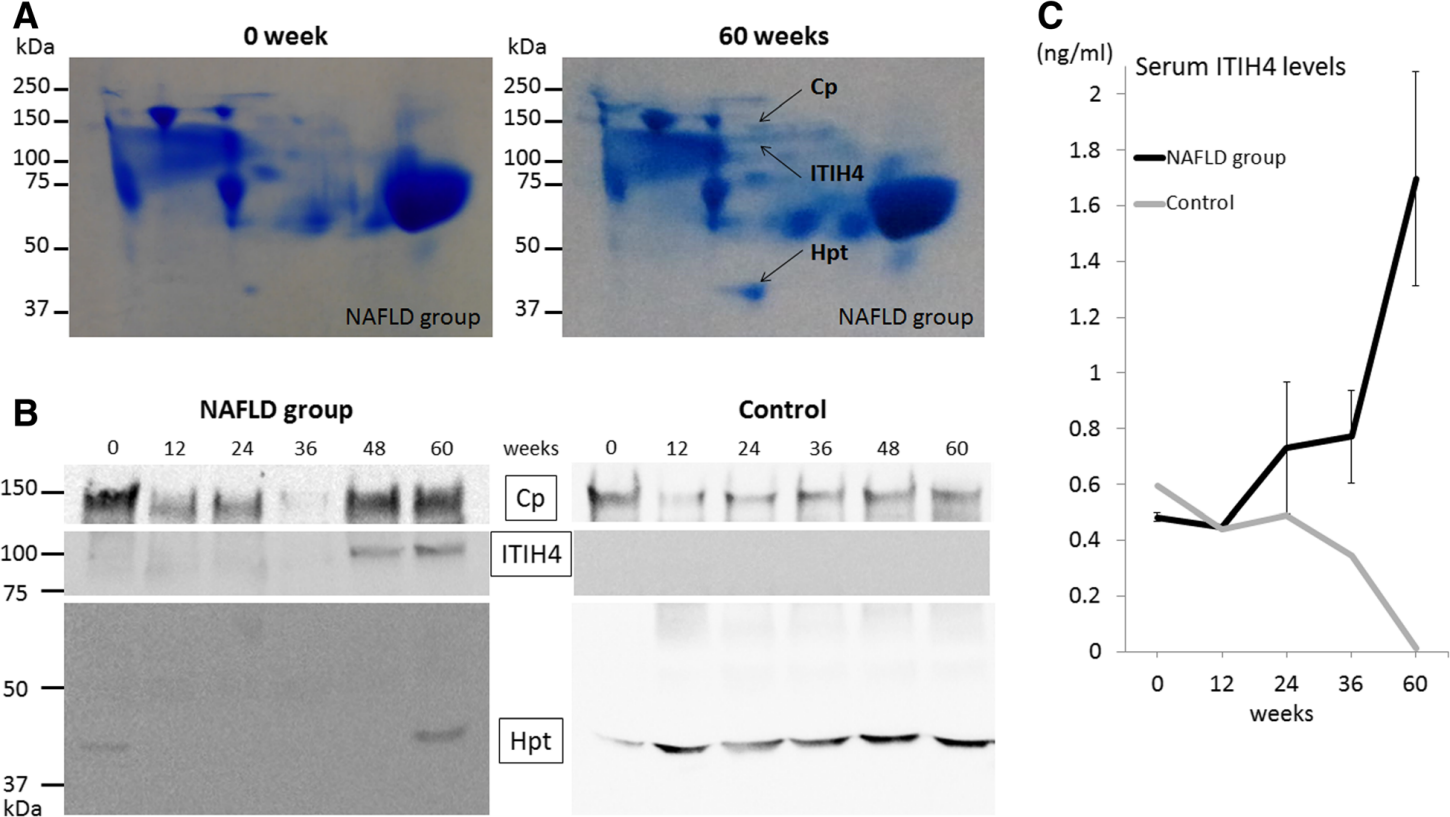
Protein identifications from the BN/SDS 2D-PAGE gels by MALDI-TOF MS/MS and validation for serum ITIH4, ceruloplasmin, and haptoglobin. **a** ITIH4, ceruloplasmin (Cp), and haptoglobin (Hpt) were identified from protein spots on a vertical line in the NAFLD group from the BN/SDS 2D-PAGE gels by MALDI-TOF MS/MS. **b** Chronological changes of serum expression of ITIH4, ceruloplasmin, and haptoglobin in the NAFLD group and control animal by western blotting. Cropped blots are showed in the figure. Full-length blots are presented in Additional file 4: Figure S3. **c** Chronological changes of serum ITIH4 levels by enzyme-linked immunosorbent assay. ITIH4 levels in the NAFLD group are expressed as the mean values. The mean values for 0, 12, 24, 36, and 60 weeks in the NAFLD group and the control were 0.48, 0.45, 0.73, 0.77, and 1.70 ng/ml, and 0.60, 0.44, 0.49, 0.34, and 0.01 ng/ml, respectively. Inter-α-trypsin inhibitor heavy chain 4: ITIH4, ceruloplasmin: Cp, haptoglobin: Hpt

In 2D BN/SDS-PAGE, serum proteins that formed an MPC were evident on one vertical gel line. Thus, we focused on the one vertical line where the expression levels of several protein spots changed over time in the NAFLD group. MALDI-TOF MS/MS on this vertical line identified ITIH4, Cp, and Hpt (Fig. 3a). In the NAFLD group, the protein spots of ITIH4, Cp, and Hpt were highly expressed at 60 weeks compared with that at 0 weeks. We conducted western blotting to validate the expression patterns of these proteins over time in both groups.

### Serum ITIH4 levels are increased in the NAFLD group

Western blotting (Fig. 3b) revealed that ITIH4 was overexpressed at 48 and 60 weeks in the NAFLD group. In the control animal, expression of ITIH4 was not detected throughout the experiment. However, the expression of Cp was higher at 48 and 60 weeks in the NAFLD group and in the control animal. The expression of Hpt was higher at 0 and 60 weeks in the NAFLD group. Based on these results, we performed quantification of serum ITIH4 by ELISA (Fig. 3c). The mean ITIH4 levels in the NAFLD group at 0, 12, 36, and 60 weeks were 0.48, 0.45, 0.77, and 1.70 ng/ml, respectively. In contrast, the ITIH4 levels in the control animal decreased to < 0.30 ng/ml at 60 weeks.

### Higher serum ITIH4 levels are associated with high NAS and HCC presence

**Fig. 4:**
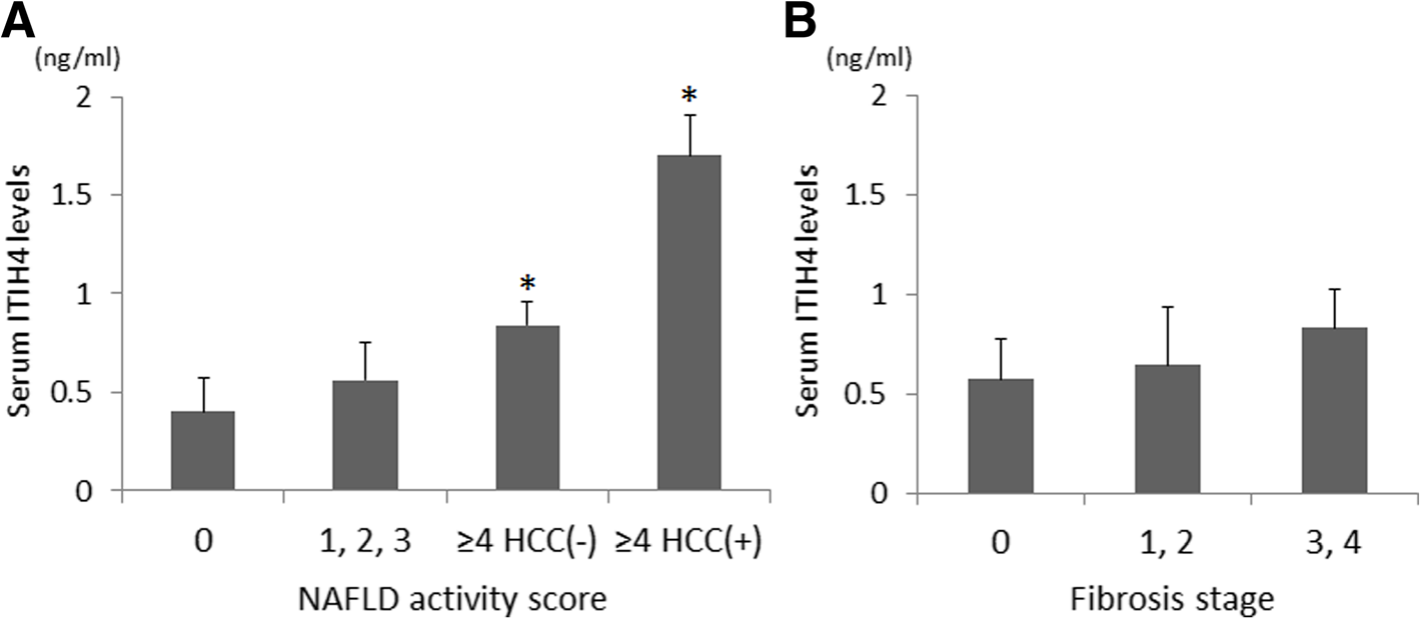
Relationship between serum ITIH4 levels and NAFLD activity scores (NAS). **a** Comparison of serum ITIH4 levels according to NAS [14] and the presence of HCC. * *P* <  0.05 compared with NAS = 0. **b** Comparison of serum ITIH4 levels according to fibrosis stage. There was no significant difference between fibrosis stages

In order to analyze the relationship between serum ITIH4 levels and histological NAS, we compared the mean serum ITIH4 levels across the four groups of samples stratified by NAS and the presence of HCC (Fig. 4a). There was no significant difference between the NAS = 0 and NAS = 1–3 groups (*P* = 0.24). ITIH4 levels in the NAS ≥ 4 without HCC group were two-fold higher than that in the NAS = 0 group, and levels in the NAS ≥ 4 with HCC group were four-fold higher (*P* = 0.024 and *P* = 0.002, respectively). In contrast, ITIH4 levels were not significantly associated with fibrosis progression (Fig. 4b).

### Hepatic ITIH4 expression at protein level shown by immunohistochemistry is upregulated in the NAFLD group

**Fig. 5:**
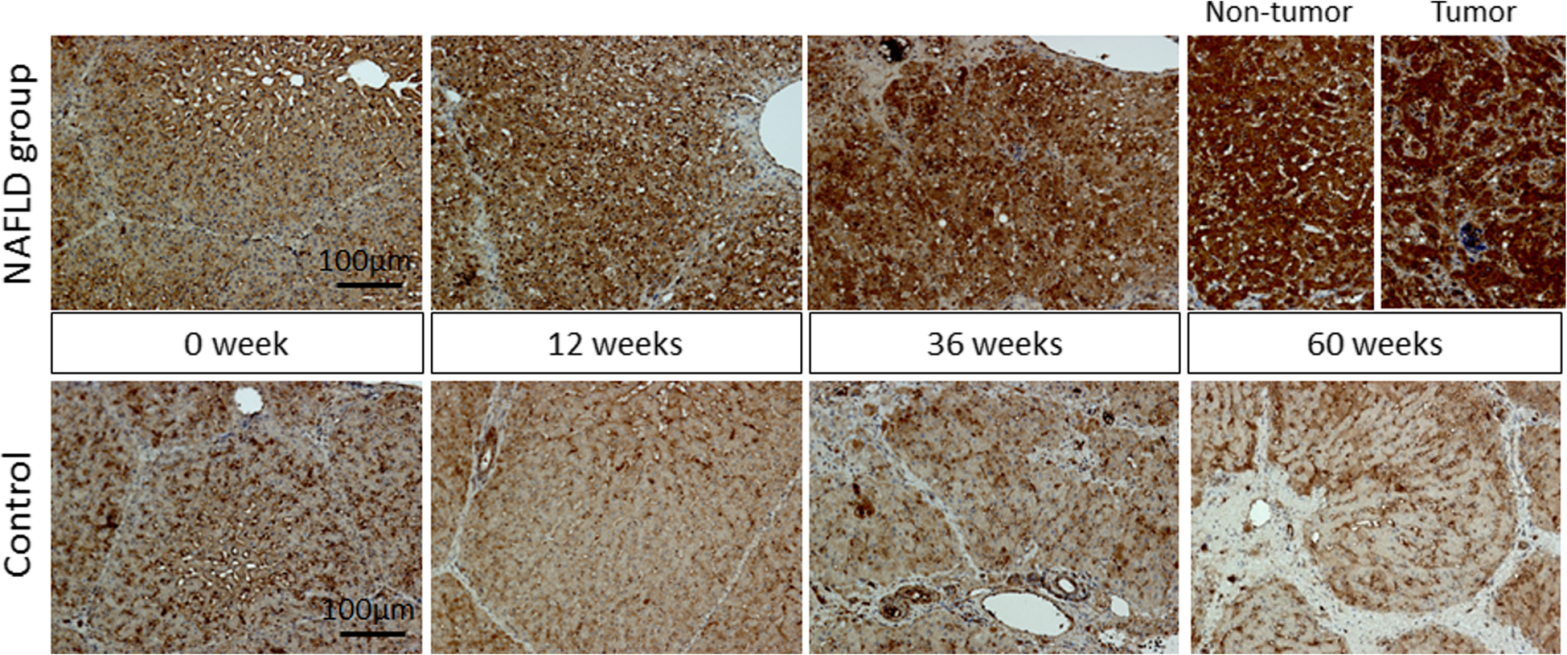
Hepatic expression of ITIH4 in the NAFLD group and control. Changes in ITIH4 immunoreactivity in the liver of both groups over time (× 200)

According to immunohistochemistry, ITIH4 staining of the cytoplasm of hepatocytes gradually increased from 0 to 60 weeks in the NAFLD group. Immunoreactivity in tumor lesions was as high as that in non-tumor lesions. In the control animal, cytoplasmic staining was faint and did not change despite the progression of fibrosis (Fig. 5).

### Serum ITIH4 levels are elevated in HCC with NAFLD patients

**Fig. 6:**
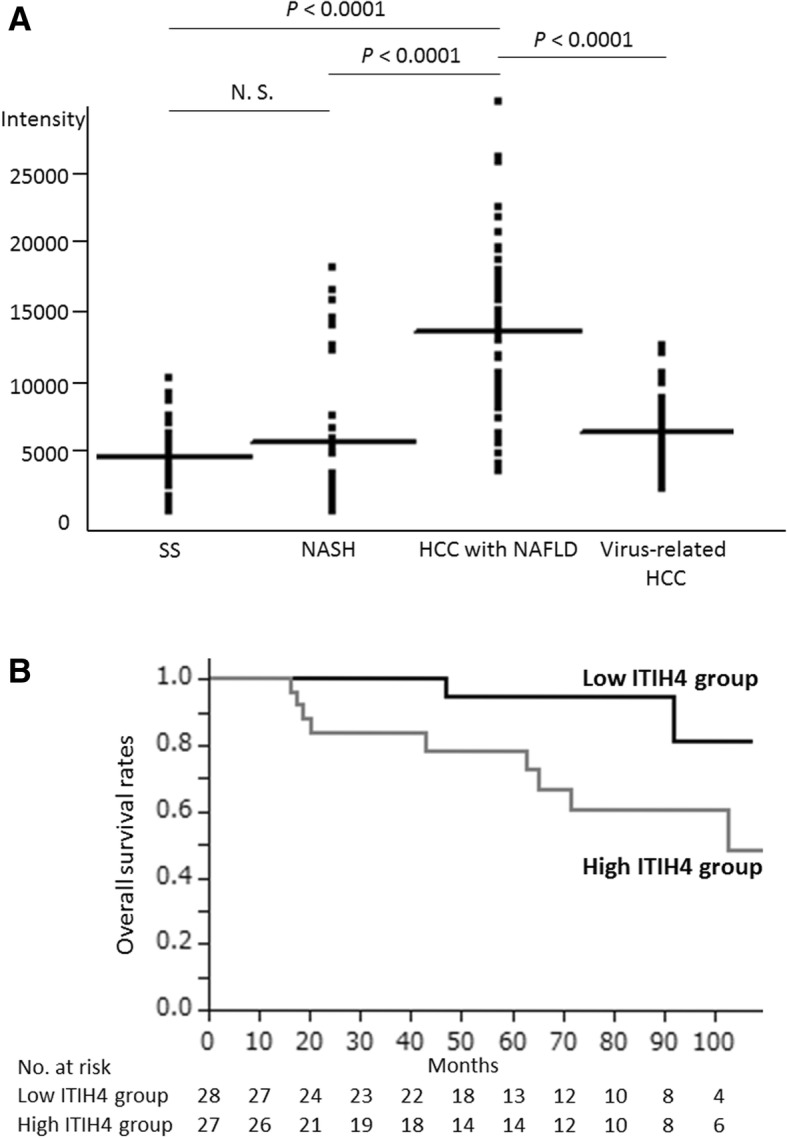
Validation for serum expression of ITIH4 in patients. **a** Serum ITIH4 intensity in the simple steatosis (SS), NASH, HCC with NAFLD, and virus-related HCC groups from western blotting quantified by densitometry. SS group: *n* = 40, NASH group: *n* = 40, HCC with NAFLD group: *n* = 55, virus-related HCC group: *n* = 35. Not significant: N.S. **b** The probability of overall survival in NAFLD with HCC patients according to serum ITIH4 intensity. The 55 patients in the HCC with NAFLD group were divided into low ITIH4 and high ITIH4 groups. The low ITIH4 and high ITIH4 groups were defined as serum ITIH4 intensity < 14,000 and serum ITIH4 intensity ≥14,000, respectively. Overall survival (OS) was defined as the period from the day of hepatectomy for HCC until the day of death caused by HCC or liver failure. For patients who survived, the date of the last follow-up was set as March 31, 2017. Cumulative 5-year overall survival rates of patients in the low ITIH4 and high ITIH4 groups were 95.0 and 72.5%, respectively (*P* = 0.0353: Log-rank, *P* = 0.0222: Wilcoxon)

In the HCC with NAFLD group, the proportion of patients who exhibited diabetes and hypertension was significantly higher than that in the SS and NASH group. In terms of liver fibrosis, the HCC with NAFLD patients exhibited reduced severity compared to the virus-related HCC patients (Table 2). Western blotting revealed that serum ITIH4 expression in the HCC with NAFLD group was significantly higher than that in the SS, NASH, and virus-related HCC groups (*P* <  0.0001). The NASH group tended to have higher expression than the SS group and 10 patients in the NASH group had > 10,000 intensities of ITIH4 expression (Fig. 6a). In the subgroup analyses that were adjusted according to the clinicopathological characteristics including fibrosis severity, the serum ITIH4 expression in the HCC with NAFLD group was significantly higher than that in the NASH group (Table 3). The area under the ROC curve of serum ITIH4 between SS and HCC with NAFLD and between NASH and HCC with NAFLD was 0.93 and 0.84, respectively (Additional file 2: Figure S2). The best cutoff point of ITIH4 intensity to distinguish the HCC with NAFLD from NASH patients was 7559. At this cut-off point, the sensitivity and specificity were 0.86 and 0.75, respectively.

**Table 2:** Clinicopathological characteristics in the SS, NASH, HCC with NAFLD, and virus-related HCC groups

	SS (*n* = 40)	NASH (*n* = 40)	HCC with NAFLD (*n* = 55)	Virus-related HCC (*n* = 35)
Gender (male)	18 (45.0%)	23 (57.5%)	43 (75.7%) * **	24 (68.6%)
Age	43 (21–67)	44 (15–76)	73 (56–90) * **	68(41–80) ***
Obesity	9 (22.5%)	26 (65.0%) *	25 (45.5%) *	10 (28.6%)
Diabetes	1 (2.5%)	5 (12.5%)	31 (56.4%) * **	7 (20.0%) ***
Hypertension	3 (7.5%)	8 (20.0%)	23 (41.8%) * **	14 (40.0%)
Child-Pugh score	5.0 (±0.1)	5.1 (±0.1) *	5.4 (±0.2) * **	5.4 (±0.2)
Fibrosis ^a^ (F3, 4)	0 (0%)	5 (12.5%) *	19 (35.9%) * **	25 (73.5%) ***
Stage ^b^ (III, IV)			22 (40.0%)	12 (34.3%)
Tumor size			5.3 (±0.5)	3.2 (±0.5) ***

Obesity was defined as a body mass index > 25

Data are expressed as *n* (%) or the mean ± standard deviation. Age is median (range)

* *P* <  0.05 compared with the SS group

** *P* <  0.05 compared with the NASH group

*** *P* <  0.05 compared with the NAFLD with HCC group

^a^ Liver fibrosis was classified according to the METAVIR scoring system [15]

^b^ Stages were categorized according to the Japanese tumor-node-metastasis staging system [16]

**Table 3:** Serum ITIH4 intensity of patients in subgroup analyses in terms of clinicopathological characteristics

Subgroup	NASH group	HCC with NAFLD group	*p* value
Patients without diabetes	4935 ± 903 (*n* = 35)	12,759 ± 1090 (*n* = 24)	< 0.0001
Patients without hypertension	4764 ± 1022 (*n* = 32)	13,228 ± 1022 (*n* = 32)	< 0.0001
Patients without obesity	5685 ± 1605 (*n* = 14)	13,772 ± 1115 (*n* = 29)	0.0002
Patients with Child-Pugh score: 5 or 6	5274 ± 917 (*n* = 40)	13,247 ± 804 (*n* = 52)	< 0.0001
Patients with F0–2 fibrosis ^a^	4809 ± 791 (*n* = 35)	14,111 ± 780 (*n* = 36)	< 0.0001

The serum ITIH4 intensity was compared between NASH and HCC with NAFLD groups

Obesity was defined as a body mass index > 25

Data are expressed as the mean ± standard deviation

^a^ Liver fibrosis was classified according to the METAVIR scoring system [15]

### Increased expression of ITIH4 are associated with poor prognosis in HCC with NAFLD patients

The high ITIH4 group exhibited larger tumor sizes than the low ITIH4 group. In terms of liver fibrosis, there was no significant difference between groups (Table 4). The 5-year OS rates of patients in the low ITIH4 and high ITIH4 groups were 95.0 and 72.5%, respectively ((*P* = 0.035: Log-rank, *P* = 0.022: Wilcoxon), Fig. 6b). In the multivariate analysis, serum ITIH4 intensity was an independent factor for OS (Additional file 3: Table S1).

**Table 4:** Clinicopathological characteristics of HCC with NAFLD patients in the low ITIH4 and high ITIH4 groups

	Low ITIH4 group (*n* = 28)	High ITIH4 group (*n* = 27)	*P* value
ITIH4 intensity	8893.7 (±699.7)	17,809.9 (±712.5)	< 0.0001
Gender (male)	21 (75.0%)	22 (81.5%)	0.56
Age	73 (56–86)	72 (59–90)	0.84
Obesity	15 (53.6%)	11 (40.7%)	0.22
Diabetes	16 (57.1%)	15 (56.6%)	0.91
Hypertension	12 (42.9%)	11 (40.7%)	0.87
Child-Pugh score	5.3 (±0.1)	5.4 (±0.1)	0.63
Fibrosis ^a^ (F3, 4)	13 (46.4%)	19 (35.9%)	0.09
Stage ^b^ (III, IV)	10 (35.7%)	12 (44.4%)	0.51
Tumor size (cm)	4.3 (±0.7)	6.3 (±0.7)	0.03
Tumor number (≥2)	11 (39.3%)	8 (29.6%)	0.45
Tumor differentiation (poorly)	6 (21.4%)	9 (33.3%)	0.31
AFP (ng/ml)	512.8 (±3232.2)	8242.5 (±3354.3)	0.10
PIVKA-II (mAU/ml)	7613 (±12,000)	25,841.6 (±12,453)	0.30
Vascular invasion (+)	4 (14.3%)	4 (14.8%)	0.96
Curability (R0 or 1)	26 (92.9%)	25 (92.6%)	0.97

The low ITIH4 and high ITIH4 groups were defined as serum ITIH4 intensity < 14,000 and serum ITIH4 intensity ≥14,000, respectively. Obesity was defined as a body mass index > 25

^a^ Liver fibrosis was classified according to the METAVIR scoring system [15]

^b^ Stages were categorized according to the Japanese tumor-node-metastasis staging system [46]

### Serum fragment of cleaved ITIH4 altered in NAFLD patients

**Fig. 7:**
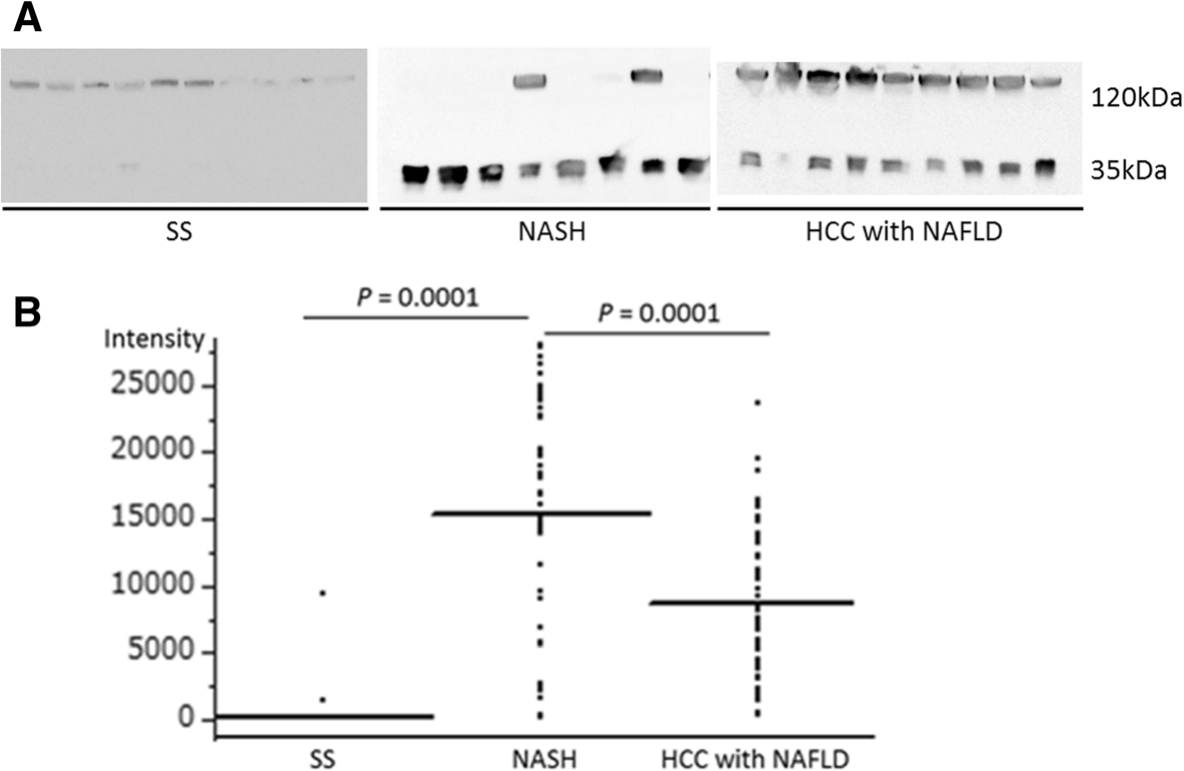
Serum expression of 35 kDa fragment of ITIH4 in patients by western blotting. **a** Expression of full-length 120 kDa and 35 kDa fragment of ITIH4 in representative blot of the SS, NASH, HCC with NAFLD group. **b** Serum 35 kDa fragment intensity in the simple steatosis (SS), NASH, and HCC with NAFLD groups from western blotting quantified by densitometry. SS group: *n* = 40, NASH group: *n* = 40, HCC with NAFLD group: *n* = 55

Serum ITIH4 is cleaved to a 35 kDa C-terminal and 85 kDa N-terminal fragments. Detection of full-length 120 kDa and 35 kDa fragment of ITIH4 in representative blot of the SS, NASH, HCC with NAFLD group were shown in Fig. 7a. In the western blotting, serum expression of 35 kDa fragment in the NASH group was significantly upregulated than that in the SS and HCC with NAFLD groups ((*P* = 0.0001), Fig. 7b).

## Discussion

To the best of our knowledge, our study is the first report of a pig model replicating HCC associated with NAFLD. Serum proteomics on swine HCC with NAFLD model implicated ITIH4 as a non-invasive biomarker reflecting NAFLD progression as well as subsequent HCC development. Most importantly, the results in the swine study have been validated in human cohort studies; human ITIH4 in the sera were significantly elevated in patients with HCC with NAFLD.

An HCC pig model associated with NAFLD has not been reported previously. Our pigs were fed an originally formulated HFD containing a higher percentage of fructose that induces hepatic lobular inflammation [17], 2% cholesterol, and 0.7% sodium cholate, which are traditionally present in an atherogenic diet. As a result, animals exhibited obesity, hypertension, hyperglycemia, dyslipidemia, and atherosclerosis. Based on these systemic phenotypes, their livers showed histological features mimicking those of human NAFLD, including hepatocyte ballooning and lobular inflammation, which are mandatory features for a NASH diagnosis [18–20]. Liver tumors exhibited positive staining of GS, GPC3, HSP70, and ARG1, which are markers for human well-differentiated HCC [21, 22]. However, microvesicular steatosis was observed in the pigs; this is a less common feature (10%) in human NAFLD than macrovesicular steatosis [23]. Previous NASH pig models showed resistance to the development of macrovesicular steatosis at 24 weeks of HFD feeding [24]. While the reason for this remains unclear, the period of lipid accumulation in the liver might have an impact on the histological confirmation of steatosis. In addition, serum TG levels were not elevated in our pigs. This is consistent with the results of previous studies that used HFD-fed pig models [24–26]. In another pig model, more than 18 months of HFD feeding achieved serum triglyceride elevation [27]. Thus, longer duration studies may reveal hypertriglyceridemia.

In order to detect serum proteomic biomarkers that predict the pathological processes of HCC associated with NAFLD, we adopted a BN/SDS 2D-PAGE technique that is useful for identifying altered MPC profiles in serum as well as individual proteins involved in disease pathogenesis. In this study, ITIH4, Cp, and Hpt, which are characterized as acute phase proteins mainly secreted from the liver, were assumed to coexist in the same MPC. These proteins are mostly characterized as high density lipoprotein (HDL) associated proteome and previous studies utilizing shotgun proteomic analysis identified acute-phase response proteins in HDL as strongly implicating the lipoprotein in inflammation and the innate immune system [28, 29]. In the current MMP model, ITIH4 was found as one of the most altered proteins in that fraction especially in the late stage of disease progression. Another line of evidence supported this finding that ITIH4 is known as one of newly found BMI-associated loci, which is especially found in Asian not found in European ancestry populations [30]. This evidence is highly suggestive that ITIH4 found in MMP pig model can be utilized as a useful biomarker for Japanese population.

ITIH4 is a 120-kDa serum glycoprotein secreted primarily by the liver [31]. It is a member of the inter-alpha-trypsin chain family of proteins, a family involved in stabilization of the extracellular matrix [32, 33]. In the current pig model, serum ITIH4 levels were significantly associated with high NAS and were further elevated throughout HCC development. The significance of ITIH4 was further confirmed by immunohistochemistry of pig liver specimens, showing that it is synthesized in both cancer lesions and the parenchyma in advanced NAFLD. We also confirmed that serum ITIH4 levels were elevated according to NAFLD progression in another pig fed an HFD and administered a lower dose of DEN (20 mg/kg body weight). Our results implicate upregulated serum ITIH4 as a biomarker reflecting NAFLD progression and subsequent HCC development. In contrast, serum ITIH4 levels were not associated with the severity of fibrosis in the pigs. These results were consistent with the findings of the human serum validation experiment. Thus, serum ITIH4 levels may have clinical utility for assessing the risk of HCC development in non-cirrhotic NAFLD patients.

In the validation experiments using human samples, we evaluated the highest levels of serum ITIH4 in HCC with NAFLD patients. We also verified the diagnostic efficacy of serum ITIH4 for HCC with NAFLD. Although the optimal cut-off level of serum ITIH4 could identify the HCC with NAFLD patients from NASH patients with 86% sensitivity, several NASH patients had high levels of serum ITIH4 in spite of the absence of HCC development. In the prognostic significance of ITIH4, HCC with NAFLD patients who had preoperatively higher serum ITIH4 levels exhibited poorer prognoses after hepatectomy. Previous report showed that serum ITIH4 level was not independently associated with OS in hepatitis virus-related HCC patients [34]. Combined with our results that the ITIH4 expression in hepatocytes increased in the parenchyma of swine as NAFLD progressed, upregulation of serum 120 kDa ITIH4 may have the potential to predict HCC development from NASH, especially in the pre-cancerous state of NASH. Furthermore, we demonstrated 35 kDa fragment of serum ITIH4 was elevated in NASH patients. 120 kDa ITIH4 is cleaved by plasma kallikrein and cleaved 35 kDa fragment is assumed to remain intact [35]. Because upregulated 120 kDa ITIH4 caused by NAFLD progression was cleaved by kallikrein in serum, 35 kDa fragment might increase but 120 kDa ITIH4 might not increase in the NASH group.

Pro-inflammatory cytokines such as interleukin (IL)-6 and tumor necrosis factor alpha (TNFα) have been reported to be linked to progression from NASH to HCC [36]. Macrophage infiltration of the expanded adipose tissue that results from weight gain promotes the production and secretion of IL-6 and TNFα by adipocytes [37]. Because ITIH4 is positively regulated by IL-6 [38, 39], it is plausible that the elevation in hepatic ITIH4 synthesis is induced by long-term exposure to IL-6 originating from immune cells as well as adipocytes. Furthermore, we demonstrated that HCC patients with NAFLD exhibited higher expression of serum ITIH4 than did virus-related HCC patients. Although no report has analyzed serum ITIH4 levels in NAFLD-associated HCC patients, decreased serum ITIH4 levels have been previously reported in HCC patients with hepatitis virus-related cirrhosis [34, 40]. In our analysis, the virus-related HCC patients who had F3 or 4 fibroses showed lower expression of serum ITIH4 than the HCC with NAFLD patients with F3 or 4 fibroses (Additional file 3: Table S2). Therefore, ITIH4 appears to be associated with etiology-dependent and fibrosis-independent carcinogenesis. Higher expression of ITIH4 may be led by HCC development via persistent systemic inflammation due to obesity or metabolic syndrome, but not via complete cirrhosis caused by hepatitis virus infection. Altered expression of ITIH4 at protein level in sera was found in a number of cancers as well as obstructive pulmonary disease or bacterial and viral infections [41–45]. Although serum ITIH4 levels could be influenced by various inflammatory conditions, the fact that ITIH4 was highly synthesized in cancer lesion of NAFLD pigs to generate MPC in serum strongly supports that serum ITIH4 is closely associated with NAFLD associated HCC development. Previous report evaluated that genetically manipulated mouse model that showed activated IL-6 signaling and inactivated TGF-β signaling spontaneously developed liver tumors and steatosis [46]. In these mice, tumor formation was inhibited by downregulation of the IL-6 pathway by Itih4 deletion. Thus, ITIH4 likely plays a significant role in the pathway that is involved in TGF-β- and fibrosis-independent carcinogenesis. Because obesity and steatosis without cirrhosis has also been recognized to associate with carcinogenesis of NAFLD patients [3], ITIH4 could be a specific biomarker reflecting the pathogenesis in NAFLD as well as metabolic syndrome-related HCC.

Certain limitations and special circumstances of this study must be acknowledged. First, statistical consideration is weak in this study, because of small sample size in the pig model. By contrast, utilizing pig as a model system is advantageous because we can trace genetically matched and serial serum samples on the same experimental subject. This cannot be realized using rodent models. Second, in the current study, we adopted a blue-native gel-based proteomic approach not by shotgun proteomics. In depth proteomic analysis in combination with shotgun proteomics will be a promising strategy in understanding pathogenesis of NAFLD associated HCC in the future study.

## Conclusions

We established an HCC pig model associated with NAFLD and identified serum ITIH4 as a non-invasive biomarker reflecting NAFLD progression and subsequent HCC development. The pig model in the present study is expected to be useful for biomarker studies as well as pathological evaluation of NAFLD associated HCC. Serum ITIH4 promises to have clinical utility in monitoring NAFLD progression, and further investigations into ITIH4 function may provide a better understanding of the mechanism of HCC development in NAFLD.

## Additional files

Additional file 1: **Figure S1.** Macroscopic features of the abdominal aorta in the NAFLD group.All animals in the NAFLD group developed longitudinal atherosclerotic lesions in the abdominal aorta at 60 weeks (arrows). (TIF 942 kb)

Additional file 2: **Figure S2.** Diagnostic performance of ITIH4 intensity in circulation was evaluated by multivariate receiver operating characteristic (ROC). (a) The area under the ROC curve (AUROC) of serum ITIH4 between simple steatosis (SS) and HCC with NAFLD was 0.927. The best cutoff point of ITIH4 intensity was 8718. (b) The AUROC of serum ITIH4 between NASH and HCC with NAFLD was 0.836. The best cutoff point of ITIH4 intensity was 7559. (TIF 320 kb)

Additional file 3: **Table S1.** Univariate and multivariate analyses for overall survival of HCC with NAFLD patients. **Table S2.** Serum ITIH4 intensity of patients in subgroup analysis in terms of the fibrosis severity. (DOCX 33 kb)

Additional file 4: **Figure S3.** Full-length blots of serum ITIH4 in the NAFLD group and control animal by western blotting.Transferrin was rum on the same gels as a loading control.Inter-α-trypsin inhibitor heavy chain 4: ITIH4. (TIF 1655 kb)

## Data Availability

All data are available without restriction. Researchers can obtain data by contacting the corresponding author.

## Abbreviations

2D BN/SDS-PAGE: Two-dimensional blue native/SDS gel electrophoresis
ARG1: Arginase-1
Cp: Ceruloplasmin
DEN: Diethylnitrosamine
ELISA: Enzyme-linked immunosorbent assay
GPC3: Glypican-3
GS: Glutamine synthetase
HCC: Hepatocellular carcinoma
HCV: Hepatitis C virus
HDL-C: High-density lipoprotein cholesterol
HFD: High-fat diet
Hpt: Haptoglobin
HSP70: Heat-shock-protein 70
IL-6: Interleukin 6
ITIH4: Inter-alpha-trypsin inhibitor heavy chain 4
LDL-C: Low-density lipoprotein cholesterol
MALDI-TOF MS: Matrix-assisted laser desorption/ionization time-of-flight mass spectrometry
MCP: Multiple-protein complex
NAFLD: Nonalcoholic fatty liver disease
NAS: NAFLD activity scores
NASH: Nonalcoholic steatohepatitis
SDS: Sodium dodecyl sulfate
TC: Total cholesterol
TG: Triglycerides
TNFα: Tumor necrosis factor alpha
