# Supplementary figures and images for: Elevated levels of circulating ITIH4 are associated with hepatocellular carcinoma with nonalcoholic fatty liver disease: from pig model to human study
Source: BMC Cancer. 2019 Jun 25;19:621. doi: 10.1186/s12885-019-5825-8 (PMC6591942; doi:10.1186/s12885-019-5825-8)

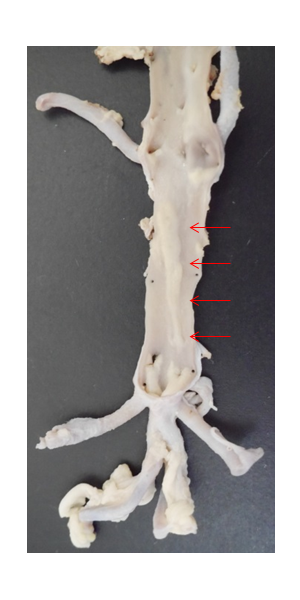

Supplement: Supplementary file 1 — Figure S1. Macroscopic features of the abdominal aorta in the NAFLD group.All animals in the NAFLD group developed longitudinal atherosclerotic lesions in the abdominal aorta at 60 weeks (arrows). (TIF 942 kb) [file 12885_2019_5825_MOESM1_ESM.tif]

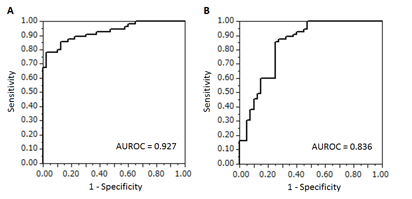

Supplement: Supplementary file 2 — Figure S2. Diagnostic performance of ITIH4 intensity in circulation was evaluated by multivariate receiver operating characteristic (ROC). (a) The area under the ROC curve (AUROC) of serum ITIH4 between simple steatosis (SS) and HCC with NAFLD was 0.927. The best cutoff point of ITIH4 intensity was 8718. (b) The AUROC of serum ITIH4 between NASH and HCC with NAFLD was 0.836. The best cutoff point of ITIH4 intensity was 7559. (TIF 320 kb) [file 12885_2019_5825_MOESM2_ESM.tif]

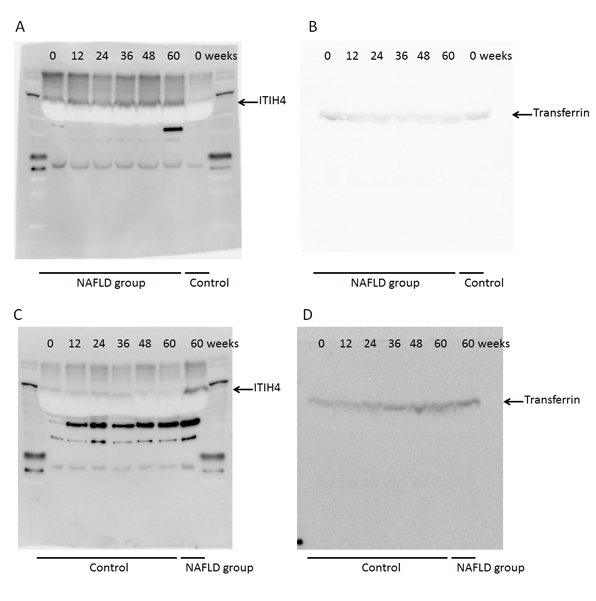

Supplement: Supplementary file 4 — Figure S3. Full-length blots of serum ITIH4 in the NAFLD group and control animal by western blotting.Transferrin was rum on the same gels as a loading control.Inter-α-trypsin inhibitor heavy chain 4: ITIH4. (TIF 1655 kb) [file 12885_2019_5825_MOESM4_ESM.tif]
