# Supplementary material for: Elevated levels of circulating ITIH4 are associated with hepatocellular carcinoma with nonalcoholic fatty liver disease: from pig model to human study
Source: BMC Cancer. 2019 Jun 25;19:621. doi: 10.1186/s12885-019-5825-8 (PMC6591942; doi:10.1186/s12885-019-5825-8)
Supplement: Supplementary file 3 — Table S1. Univariate and multivariate analyses for overall survival of HCC with NAFLD patients. Table S2. Serum ITIH4 intensity of patients in subgroup analysis in terms of the fibrosis severity. (DOCX 33 kb) [file 12885_2019_5825_MOESM3_ESM.docx]

**Supplementary Tables**

Table S1 Univariate and multivariate analyses for overall survival of HCC with NAFLD patients

|  | Univariate analysis | |  | Multivariate analysis | |
| --- | --- | --- | --- | --- | --- |
|  | HR | *P* value |  | HR | *P* value |
| Age (≥75/<75) | 1.43 | 0.56 |  |  |  |
| Gender (male/female) | 1.42 | 0.72 |  |  |  |
| Obesity (+/-) | 0.42 | 0.17 |  |  |  |
| Diabetes (+/-) | 0.91 | 0.88 |  |  |  |
| Hypertension (+/-) | 1.21 | 0.77 |  |  |  |
| Child-Pugh score (≤6/>6) | 0.97 | 0.98 |  |  |  |
| Fibrosis ᵃ (F3, 4/F0, 1, 2) | 2.10 | 0.06 |  | 6.28 | 0.01 |
| Tumor size (≥5cm/<5cm) | 10.94 | 0.002 |  | 19.86 | 0.0003 |
| Tumor number (≥2/<2) | 1.84 | 0.33 |  |  |  |
| Tumor differentiation (poorly/others) | 0.52 | 0.37 |  |  |  |
| Vascular invasion (+/-) | 0.68 | 0.70 |  |  |  |
| Curability ᵇ (R0 or 1/R2) | 0.96 | 0.97 |  |  |  |
| serum ITIH4 intensity (≥14000/<14000) | 4.53 | 0.02 |  | 4.55 | 0.03 |

Statistically significant *p* values and hazard ratios (HR) are shown.

Factors with *p* value < 0.1 in the univariate analyses were included into the multivariate analyses.

Obesity was defined as a body mass index > 25.

ᵃ Liver fibrosis was classified according to the METAVIR scoring system [45].

ᵇ Curability were categorized according to the Japanese tumor-node-metastasis staging system [46].

Table S2 Serum ITIH4 intensity of patients in subgroup analysis in terms of the fibrosis severity

| Patients with F3, 4 fibrosis | Virus-related HCC (*n* = 25) | HCC with NAFLD (*n* = 19) | *p* value |
| --- | --- | --- | --- |
| Serum ITIH4 intensity | 5953.4±911.8 | 11678.3±1045.9 | 0.0002 |

The serum ITIH4 intensity was compared between virus-related HCC and NAFLD with HCC groups among the patients with F3 or F4 fibrosis [45].

Data are expressed as the mean ± standard deviation.
